# Optic radiations are thinner and show signs of iron deposition in patients with long-standing remitting-relapsing multiple sclerosis: an enhanced T_2_^*^-weighted angiography imaging study

**DOI:** 10.1007/s00330-018-5461-8

**Published:** 2018-04-30

**Authors:** Chun Zeng, Silin Du, Yongliang Han, Jialiang Fu, Qi Luo, Yayun Xiang, Xiaoya Chen, Tianyou Luo, Yongmei Li, Yineng Zheng

**Affiliations:** Department of Radiology, The First Affiliated Hospital of Chongqing Medical University, No. 1 Youyi Road, Yuzhong District, Chongqing, 400016 China

**Keywords:** Demyelinating disease, Multiple sclerosis, Magnetic resonance imaging, White matter, Iron

## Abstract

**Objective:**

This study aimed to investigate iron deposition and thickness and signal changes in optic radiation (OR) by enhanced T_2_^*^-weighted angiography imaging (ESWAN) in patients with relapsing-remitting multiple sclerosis (RRMS) with unilateral and bilateral lesions or no lesions.

**Methods:**

Fifty-one RRMS patients (42 patients with a disease duration [DD] ≥ 2 years [group Mor], nine patients with a DD < 2 years [group Les]) and 51 healthy controls (group Con) underwent conventional magnetic resonance imaging (MRI) and ESWAN at 3.0 T. The mean phase value (MPV) of the OR was measured on the phase image, and thickness and signal changes of the OR were observed on the magnitude image.

**Results:**

The average MPVs for the OR were 1,981.55 ± 7.75 in group Mor, 1,998.45 ± 2.01 in group Les, and 2,000.48 ± 5.53 in group Con. In group Mor, 28 patients with bilateral OR lesions showed bilateral OR thinning with a heterogeneous signal, and 14 patients with unilateral OR lesions showed ipsilateral OR thinning with a heterogeneous signal. In the remaining nine patients without OR lesions and in group Con, the bilateral OR had a normal appearance. In the patients, a negative correlation was found between DD and OR thickness and a positive correlation was found between MPV and OR thickness.

**Conclusions:**

We confirmed iron deposition in the OR in the RRMS patients, and the OR thickness was lower in the patients than in the controls.

**Key Points:**

*• Enhanced T*_2_^*^ *-weighted magnetic resonance angiography (ESWAN) provides new insights into multiple sclerosis (MS).*

*• Focal destruction of the optic radiation (OR) is detectable by ESWAN.*

*• Iron deposition in OR can be measured on ESWAN phase image in MS patients.*

*• OR thickness was lower in the patients than in the controls.*

*• Iron deposition and thickness changes of the OR are associated with disease duration.*

**Electronic supplementary material:**

The online version of this article (10.1007/s00330-018-5461-8) contains supplementary material, which is available to authorized users.

## Introduction

Multiple sclerosis (MS) is a multifactorial disease of the central nervous system (CNS) characterised by inflammation, demyelination, neuro-axonal loss, gliosis and diffuse degeneration [1–3]. Visual dysfunction is a common clinical manifestation of MS, and affects approximately 50% of patients [4–6]. Susceptibility of the visual system to damage in MS makes it an ideal model to study the pathophysiology of MS [7]. The optic radiation (OR) transmits information from the ipsilateral temporal and contralateral nasal hemiretinae, which project from the interneurons of the lateral geniculate nucleus to the striate cortex [8]. The large and highly myelinated axons pass through the periventricular white matter, and are susceptible to focal inflammatory damage in MS [8]. However, the extent of abnormalities in the OR of MS patients is not fully understood. Many explanations, such as primary OR pathology, subclinical inflammation of the OR, and retrograde degeneration, have been offered, but no convincing evidence has been produced to support any of those hypotheses.

Although conventional magnetic resonance imaging (MRI) plays a crucial role in the observation of changes in the OR [9], its contribution to understanding potential mechanisms and the relationship to pathological features is limited due to low specificity. Some studies have shown that diffusion tensor imaging (DTI) allows the examination of the OR integrity loss [10, 11], but DTI does not allow the visualisation of focal demyelination. Recently, a study reported that the OR is constantly depicted as a low-signal band (LSB) in susceptibility-weighted imaging (SWI) at 3T [12]. In a previous report, the high-spatial-resolution 3T phase difference-enhanced (PADRE) images delineated various small fibre tracts of the brainstem, such as the medial and dorsal longitudinal fascicules and the central tegmental tract, which have been difficult to visualise using conventional MRI [13]. However, information regarding the element(s) is responsible for the phase differences between the tract and surrounding structures, especially in MS remaining unclear [12–14].

Recently, enhanced T_2_^*^-weighted angiography imaging (ESWAN) was shown to combine a unique three-dimensional (3-D) T_2_^*^-based multi-echo method of acquisition with a special reconstruction algorithm [15]. ESWAN has significant advantages over conventional single-echo SWI, including a high and enhanced susceptibility-sensitive spatial resolution, a high signal-to-noise ratio and a reduced chemical shift artefact. These unique properties make ESWAN an attractive technique for imaging iron deposition on the phase image and signal and thickness changes in the OR on the magnitude image [15]. Realising the excellent visibility of the OR by using ESWAN at 3T, we aimed to retrospectively observe the characteristics of iron deposition and signal and thickness changes in the OR in patients with relapsing-remitting multiple sclerosis (RRMS).

## Materials and methods

### Subjects

We investigated 51 consecutive RRMS patients (nine patients with a disease duration [DD] < 2 years [group Les], 42 patients with a DD ≥ 2 years [group Mor]) with visual impairment and without a history of clinical optical neuritis (ON) in both eyes. RRMS was diagnosed according to the 2016 revised criteria [16]. None of these patients had been previously diagnosed with a cerebrovascular disease, other neurological diseases or cardiovascular morbidity. Additionally, none of the patients had a contraindication for having a contrast agent injection as part of an MRI examination. The patients were recruited from the Neurological Department of the First Affiliated Hospital of Chongqing Medical University (Chongqing, China) from September 2013 to August 2016. All the patients were clinically assessed on the day of the MRI using the Expanded Disability Status Scale (EDSS) [17]. Chun Zeng and Silin Du examined all the patients. For comparison, 51 consecutive healthy subjects (group Con) were enrolled in this study. According to the institutional guidelines, all subjects signed an informed written consent form approved by our institutional review board before undergoing MRI.

### MRI acquisition

All the patients underwent brain scan using a 3.0-T magnetic resonance (MR) system (GE Medical system, Milwaukee, WI, USA) and an eight-channel phased-array head coil. A standard protocol for MS studies was performed that included axial dual-echo proton density (PD)-T_2_-weighted imaging (T_2_WI), and axial fluid-attenuated inversion recovery (FLAIR). In addition, post-contrast T_1_WI was reperformed again for all patients after injecting 0.1 ml/kg body weight of gadolinium contrast agent (Bayer-Schering Pharma, Milan, Italy).

Before injecting gadolinium, 3-D ESWAN data were was acquired with eight echoes using the following parameters: repetition time (TR) = 60 ms, effective echo time (TE) = 6 ms, number of excitation (NEX) = 0.75, field of view (FOV) = 22 cm × 22 cm, matrix = 448 × 320, receiver bandwidth = +62.5 kHz, and flip angle = 20°. The sequence was acquired with 2-mm-thick contiguous sections and no space. All scans were oriented parallel to the anterior-posterior commissural (AC-PC) line with 56–64 locations on the middle sagittal plane and covered the entire brain area.

### Observation of the OR lesions

The lesions affecting the OR without enhancement were hypointense on T_1_WI and hyperintense on correponding T_2_WI and FLAIR, and were confirmed by one clinical neuroradiologist with more than 10 years of experience in clinical and scientific MS imaging (*BLINDED*), who was not aware of the paraclinical and clinical data. Virchow-Robin spaces were excluded by their tubular appearance and FLAIR hypointense.

### Image post-processing

**Fig. 1:**
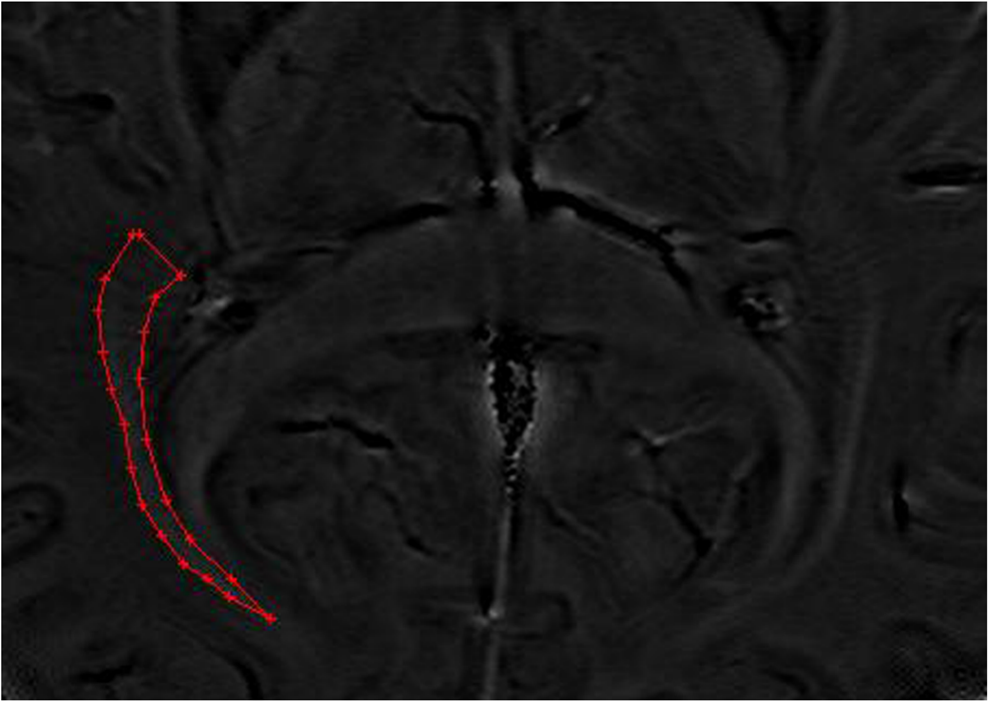
Bilateral optic radiation (OR) shows symmetrical, slightly hyperintense signals in the phase image of enhanced T_2_^*^-weighted angiography imaging (ESWAN) in a normal participant, and the region of interest (ROI) is placed in the OR for the measurement of the mean phase value (MPV)

**Fig. 2:**
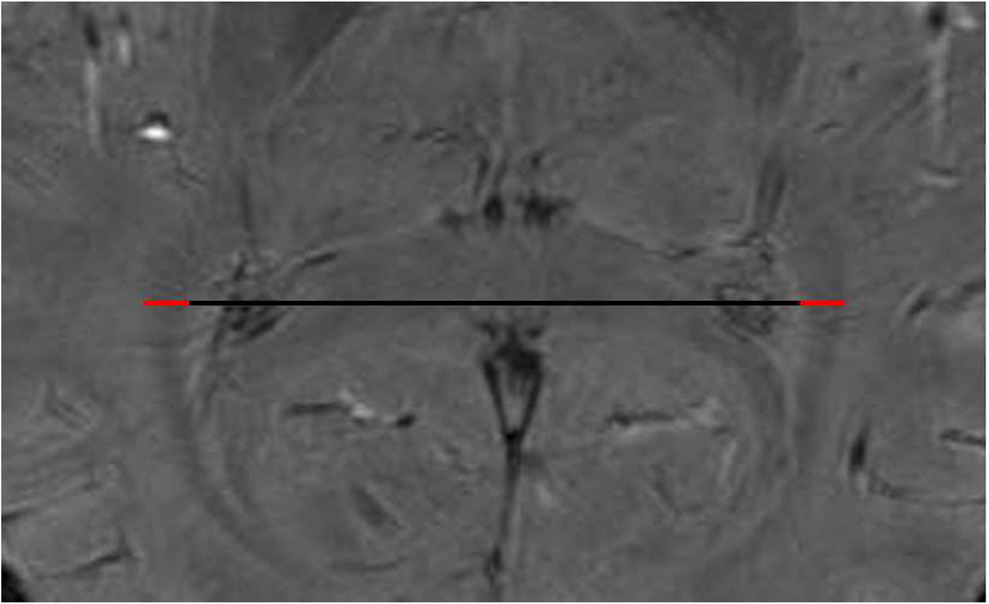
Bilateral optic radiation (OR) shows symmetrical, hypointense signals on the magnitude image in a normal participant. Thickness is measured at two positions (red lines) in each of three consecutive slices in the magnitude image, and final thickness is the mean value for the three consecutive slices

All ESWAN images were post-processed using research software (FuncTool 6.3.1 software; GE Healthcare) on an ADW4.4 workstation (Sun Microsystems, Santa Clara, CA, USA). A high-pass filter was applied in the subsequent processing steps to reduce aliasing artefacts, which arise predominantly in images involving air-tissue interfaces and background field inhomogeneities. The next step was to create a new type of phase image, which was referred to as a phase mask. Separate acquisition of phase and magnitude data allowed postprocessing to be performed offline instead of using the MRI system. Offline processing used the SPIN software (Signal Processing In NMR; http://www.mrc.wayne.edu/download). For paramagnetic substances, an increase in magnetic field led to a negative phase relative to that of the surrounding parenchyma and CSF. First, an observer adjusted the brightness and contrast of each of the phase and magnitude images to obtain improved definition of the anatomical reference structures, consequently allowing greater accuracy in the measurement. Second, the ORs were magnified to the same degree for a clearer definition of the OR margins. Third, the mean phase values (MPVs) of the OR were measured on the phase images (Fig. 1), and thicknesses were measured and signals were observed on the magnitude images (Fig. 2). For each subject, three continuous axial slices characterised by a high inter-individual comparability were selected: (a) one slice tangential to the inferior splenium of the corpus callosum and superior to the vein of Galen, (b) one slice parallel and inferior to the first slice, and (c) one slice parallel and superior to the first slice. To ensure data accuracy, all MPVs and thicknesses were the mean value for three slices [14]. All post-processing of structural MRI was performed three times with repeated measurements by an observer (Yongmei Li), and the final result was the mean value of the three measurements.

Phase images served as a direct measure of the magnetic field variation based on the following formulas: ф (phase) = -r Δ*B*TE (where *r* signifies gyromagnetic, Δ*B* is the change in the magnetic field between tissues and TE is the echo time); Δ*B* = c*V*Δ*xB*o (where c is the iron content, *V* is the voxel volume and Δ*x* is the variation in molar susceptibility between tissues. When iron is present, at a given TE, the more iron that exists in the tissue, the more the phase will decrease. Therefore, the phase contrast will depend on how much iron is present [18]. The characteristics of the OR thickness and signal were evaluated and categorised into the following three types: (1) bilateral thinning of the OR with a heterogeneous signal; (2) unilateral thinning of the OR with a heterogeneous signal; and (3) a bilateral OR with a normal thickness and homogeneous signal. MPVs and thicknesses of the OR were averaged between hemispheres.

### Statistical analysis

Statistical analysis was performed using the SPSS statistical package (SPSS for Windows, software version 17.0; SPSS, Chicago, IL, USA). One-way analysis of variance was used to assess group differences in average MPV between MS patients and healthy controls. Intergroup comparisons among group Les, group Mor and group Con were performed using a least significant difference procedure. In addition, correlation analyses were performed using Pearson’s correlation coefficient. All the tests were two-tailed (α = 0.05), and *p* < 0.05 was considered statistically significant.

## Results

Fifty-one RRMS patients and 51 corresponding healthy controls were enrolled. The demographic data are presented in Table 1.

**Table 1:** Demographic data of the patients and healthy controls

	n	Age, years	Sex, F/M	DD, years	EDSS score
Patients with a DD < 2 years (group Les)	9	29 ± 8.6	7/2	1.4 ± 0.5	3.4 ± 1.4
Patients with a DD ≥ 2 years (group Mor)	42	32.3 ± 7.7	29/13	4.4 ± 1.3	3.0 ± 1.9
Healthy controls (group Con)	51	31.1 ± 7.7	35/16	n/a	n/a

*DD* disease duration, *EDSS* Expanded Disability Status Scale

### Evaluation of the OR lesions

**Fig. 3:**
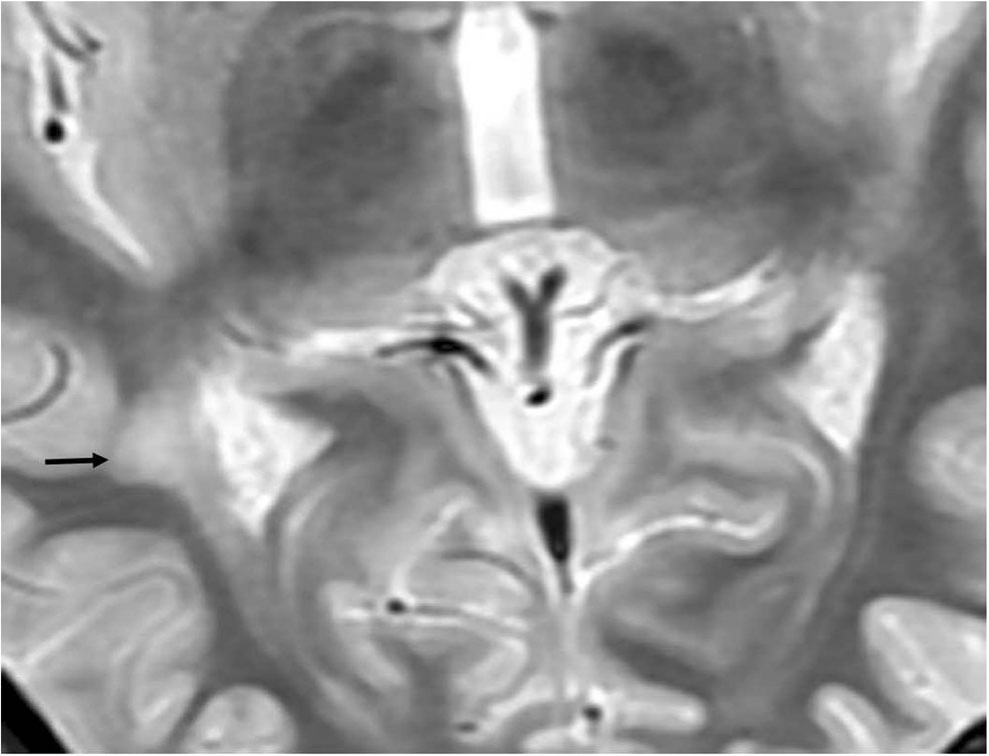
Conventional T_2_WI shows one lesion in the right optic radiation (OR) (arrow) in a 32-year-old patient with a disease duration (DD) of 1.2 years

**Fig. 4:**
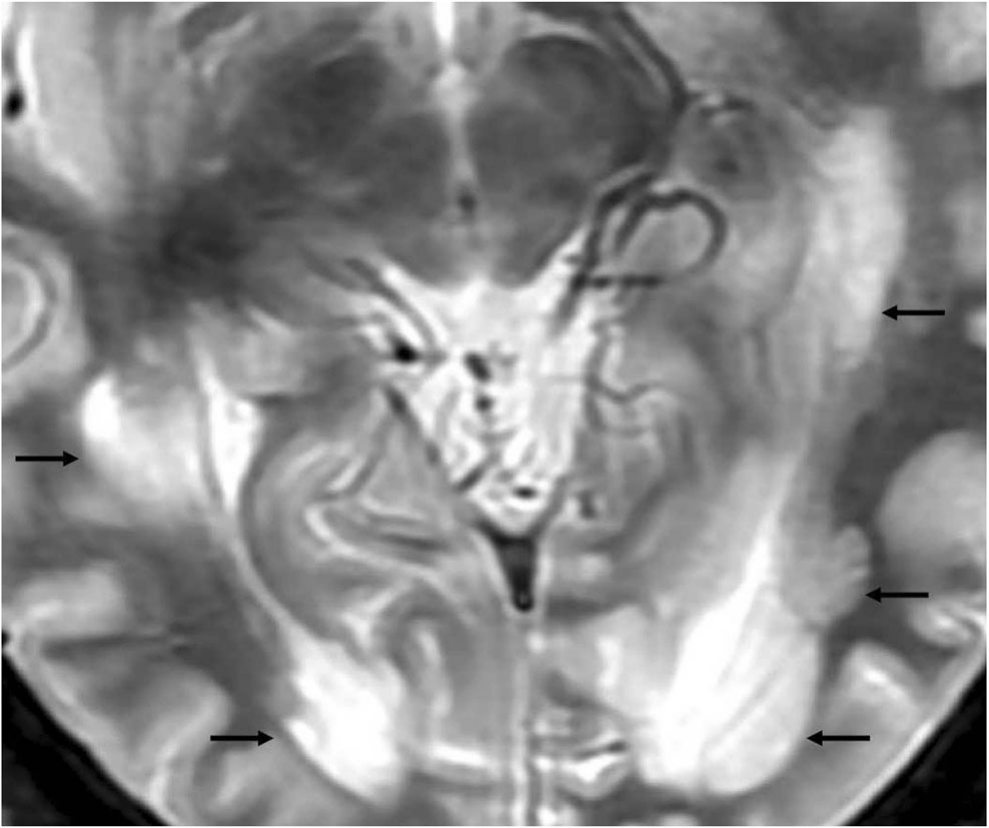
Conventional T_2_WI shows multiple lesions in both optic radiations (ORs) (arrows) in a 29-year-old patient with a disease duration (DD) of 6.5 years

One hundred and eight non-enhancing lesions, which were hypointense upon T_1_WI and hyperintense upon T_2_WI, were observed in the OR regions of 33 patients (26 patients in group Mor and seven patients in group Les). Of the 33 patients, 18 in group Mor and two in group Les had bilateral OR lesions (Fig. 3) and eight patients in group Mor and five patients in group Les had unilateral OR lesions (Fig. 4). No lesions were detected in the OR regions of the remaining 18 patients (16 patients in group Mor and two patients in group Les).

### Normal manifestation of the OR on ESWAN phase and magnitude images

In healthy controls, the OR showed bilateral symmetrical high-signal bands (HSBs) on the phase images (Fig. 1) and LSBs on the corresponding magnitude images (Fig. 2) that were lateral to the lateral ventricles.

The average MPVs for the OR were 1,981.55 ± 7.75 in group Mor, 1,998.45 ± 2.01 in group Les, and 2,000.48 ± 5.53 in group Con (Table 2). Group analysis revealed that this difference was driven by the DD of the patients. The MPV of the OR was significantly lower in group Mor than in group Les (*p* < 0.05) and group Con (*p* < 0.01), and the MPV of the OR was significantly lower in group Les than in group Con (*p* < 0.01).

**Table 2:** Average mean phase value (MPV) for optic radiation (OR) in group Mor, group Les and group Con

Group	Mean	Standard deviation	95% confidence interval for the mean	
			Lower bound	Upper bound
Group Mor	1,981.55	7.75	1,979.03	1,983.90
Group Les	1,998.45	2.01	1,997.23	2,000.12
Group Con	2,000.48	5.53	1,999.46	2,002.4

Pearson’s correlation analysis showed a remarkable negative correlation between the DD and MPVs of the OR in the RRMS patients (R = 0.91, *p* < 0.001); However, no correlation was found between the MPV and EDSS (*p* > 0.05).

### Analysis of the signal and thickness in the OR

**Fig. 5:**
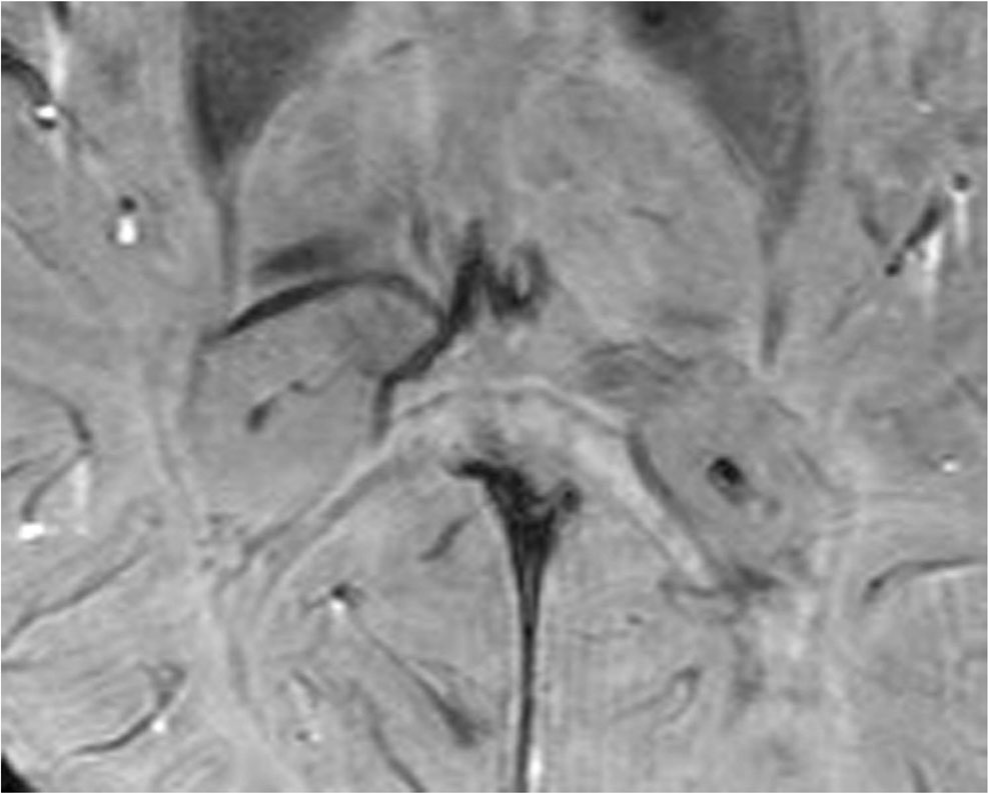
Bilateral optic radiations (ORs) are significantly thin and show heterogeneous signals in the magnitude image in a 40-year-old patient with a disease duration (DD) of 7.0 years (arrows)

**Fig. 6:**
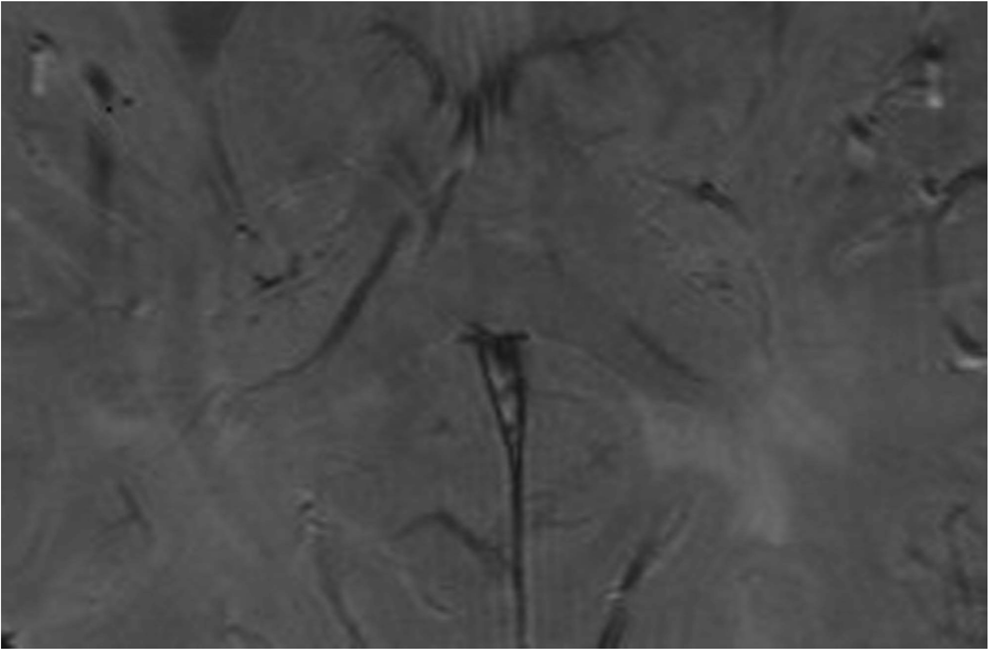
The left optic radiation (OR) with more lesions shows a vanished signal in the magnitude image of a 38-year-old patient with a disease duration (DD) of 4.6 years

In group Mor, 28 patients with bilateral OR lesions showed thinning of the bilateral OR with a heterogeneous signal (Fig. 5) and 14 patients with unilateral OR lesions showed thinning of the ipsilateral OR with a heterogeneous signal (Fig. 6). In the group Les, for the remaining nine patients without OR lesions, the bilateral OR appeared normal with a homogeneous signal.

The average thicknesses of the OR were 3.2 mm, 3.8 mm, and 3.8 mm in group Mor, group Les, and group Con, respectively. There was no significant difference in the thickness of the OR between group Con and group Les (*p* > 0.05). Additionally, the thickness of the OR was lower in group Mor than in group Con (*p* < 0.05).

Pearson’s correlation analysis showed a remarkable negative correlation between the DD and thickness of the OR in the patients (R = 0.9, *p* < 0.001); however, no correlation was found between the thickness and EDSS in RRMS patients (*p* > 0.05).

### Correlation between the MPV and thickness of the OR in RRMS patient

We found a significant positive correlation between the MPV and thickness of the OR in the patients (R = 0.93, *p* < 0.001).

## Discussion

ESWAN is a robust and powerful tool that can offer distinct measurement and visualisation of the OR. To the best of our knowledge, this is the first study to investigate characteristic iron deposition and thickness and signal changes in the OR in RRMS patients using ESWAN. This study had three main outcomes. First, we found that the average MPV in the OR decreased sequentially from group Con to group Les to group Mor. Additionally, we observed that iron deposition in the OR was correlated with the DD of the patients. Second, group Mor had the lowest OR thickness of the three groups, and there was no significant difference in the OR thickness between group Con and group Les. Pearson’s correlation analysis showed a remarkable negative correlation between the DD and thickness of the OR in the patients. Third, we observed a significant correlation between the MPV and OR thickness in the patients.

### Iron deposition in the OR in RRMS patients

Our results showed that the OR was slightly hyperintense on the phase image of ESWAN in both the patients and the controls, whereas the average MPV of the OR in the patients was lower than that in the controls. Chen et al. [19] and Du et al. [20] previously had demonstrated a negative correlation between MPV and brain iron concentration in MS patients. Thus, we speculated that a small number of iron depositions that cannot be distinguished with the naked eye may exist in the OR in patients. Recently, a study found that the phase value, which is indicative of greater iron content, was significantly higher in MS patients than in healthy controls and was associated with more severe lesion burden and brain atrophy [21]. Many studies have demonstrated increased iron deposition in the brain in MS [22, 23]. One possible source of iron deposition in the OR is macrophages, and microglia may acquire high levels of iron by phagocytosing myelin/oligodendrocyte debris.

Thus far, a few theories have been proposed to explain the relationship between iron and MS. Some researchers have considered that the breakdown of the blood-brain barrier (BBB) in MS can result in the extravasation of red blood cells into the CNS [24]. Other researchers have considered iron deposition to be only a secondary phenomenon of MS [25]. Khalil et al. showed that iron accumulation did not precede the development of MS but rather occurred as a by-product of the pathophysiological processes of the disease and was related to morphological brain damage [26]. However, the precise mechanisms underlying this excessive iron accumulation in MS are still uncertain.

In addition, we found a remarkable negative correlation between DD and MPV in the OR in patients, which was consistent with several prior studies. Zhang et al., using a 3-D T_2_^*^-weighted spoiled multi-echo GRE sequence in a 3T MRI system, demonstrated that non-enhancing lesions with a longer DD showed a higher relative susceptibility/R_2_^*^ value, reflecting iron deposition [2]. Hammond et al. demonstrated that the local field shift (LFS), which is caused by magnetic susceptibility-shifted compounds such as iron, was significantly correlated with DD in the caudate and putamen in MS [27]. Similarly, Reshiana et al. demonstrated that precentral grey matter (GM) MPVs were negatively correlated with DD [21].

Our study found no correlation between MPVs and EDSS scores. This result can be partially explained by the small amount of iron deposition that may not cause immediate effects and are not reflected in the EDSS score. Another possible reason is that the OR is part of the visual system but EDSS is a measure of physical disability in MS and is heavily weighted toward motor disability. Similarly, Khalil et al. found that short-term changes in iron concentration were not associated with changes in disability in 76 patients with clinically isolated syndrome (CIS) and 68 patients with MS [23], and Du et al. found no correlation between the MPVs of all regions of interest (ROIs) and EDSS scores in the study. Conversely, Rudko et al. showed that quantitative susceptibility (QS) maps more greatly aided the identification of significant, voxel-level increases in iron deposition in the subcortical GM of MS patients compared with control subjects. ROI analysis of the mean R_2_^*^ and QS in the subcortical GM demonstrated that R_2_^*^ and QS were strongly correlated with EDSS [28]. We considered that discrepancy may be due to differences in the population characteristic and region in anatomy.

### Thickness changes in the OR in RRMS patients

Our results showed that the bilateral OR around the trigone of the lateral ventricle was hypointense, which is consistent with other reports [12, 14]. A histological analysis of a post-mortem specimen confirmed that the identified T_2_^*^W-hypointense periventricular structures reflect the OR [13]. Moreover, we demonstrated that patients with a longer DD had a thinner OR than controls. A plausible explanation is focal axonal trans-section within MS plaques after a long DD [13, 29]. Daniel et al. demonstrated that the median MRI indices along the OR were also significantly abnormal in all MS subgroups [30]. Interestingly, 28 patients with bilateral OR lesions showed bilateral thinning of the OR and a heterogeneous signal, and 14 patients with unilateral OR lesions showed homolateral thinning of the OR and a heterogeneous signal. Consistent with the results of a previous study [4], the analysis of DTI demonstrated that T_2_ OR+ patients had a more severe and distributed pattern of DTI abnormalities along the ORs than did healthy controls and T_2_ OR- patients. The notion is that trans-synaptic retrograde degeneration of the OR due to MS lesions might occur in these patients.

Surprisingly, in the nine patients with a shorter DD, the OR had a normal thickness and homogeneous signal. However, these nine patients had a lower MPV than the controls, possibly because the iron accumulation in the OR is more pronounced in early stages and occurs independent of its morphology. Chen et al. demonstrated that the fractional anisotropy (FA) values were significantly lower, and the mean diffusivity (MD), λ_∥_, λ_⊥_values were significantly higher in the unaffected OR in MS patients than in controls. Similarly, using 3D-ESWAN, Zeng et al., observed that the ICVs and their main tributaries had a score of 3, with the presence of continuous vessel walls and homogeneous signals in MS patients with a relatively short DD.

### Limitations

Although we believe that our findings in this study are substantial and have a good pathophysiological correlation, they are not without limitations. First, the cross-sectional design and relatively small number of subjects studied may not provide an accurate reflection of the iron deposition and thinning of the OR in MS patients. Second, owing to the nature of ESWAN phase images and the associated ‘blooming’ effects, our measurements of iron are inherently relative rather than absolute. Third, OR quantification may be influenced by MS lesions; however, the assessment of the correlation between OR damage and the severity of clinical involvement has been a topic of previous investigation and is beyond the scope of our study.

## Conclusion

3D-ESWAN can detect iron deposition in the OR in RRMS patients with visual impairment but without ON from the early stage of the disease onwards, and the thickness of the OR in patients with a longer DD is lower than that in the controls. Thus, iron deposition in the OR is more pronounced in the early stage and occurs independent of its morphology. Furthermore, the DD was found to be a significant parameter in patients with RRMS. Further studies are needed to confirm and elucidate these findings.

## Electronic supplementary material

ESM 1 (DOCX 46 kb)

## Abbreviations

BBB: Blood-brain barrier
CNS: Central nervous system
DD: Disease duration
DTI: Diffusion tensor imaging
ESWAN: Enhanced T_2_^*^-weighted angiography imaging
FA: Fractional anisotropy
HSB: High-signal band
LSB: Low-signal band
MPV: Mean phase value
MRI: Magnetic resonance imaging
MS: Multiple sclerosis
ON: Optical neuritis
OR: Optic radiation
PADRE: Phase difference enhanced
RRMS: Relapsing-remitting multiple sclerosis
SWI: Susceptibility-weighted imaging
