# Supplementary material for: Optic radiations are thinner and show signs of iron deposition in patients with long-standing remitting-relapsing multiple sclerosis: an enhanced T2*-weighted angiography imaging study
Source: Eur Radiol. 2018 Apr 30;28(10):4447–54. doi: 10.1007/s00330-018-5461-8 (PMC6132724; doi:10.1007/s00330-018-5461-8)
Supplement: Supplementary file 1 — (DOCX 46 kb) [file 330_2018_5461_MOESM1_ESM.docx]

Correlation between the DD and MPVs of the OR in the RRMS patients (R=0.91, P < 0.001).

Correlation between the DD and thickness of the OR in the RRMS patients (R = -0.91, P < 0.001).

Correlation between the MPV and thickness of the OR in the RRMS patients (R=0.93, P < 0.001).
